# Near-field sensor array with 65-GHz CMOS oscillators can rapidly and comprehensively evaluate drug susceptibility of *Mycobacterium*

**DOI:** 10.1038/s41598-023-30873-9

**Published:** 2023-03-07

**Authors:** Shojiro Kikuchi, Yoshihisa Yamashige, Ryosuke Hosoki, Masahiko Harata, Yuichi Ogawa

**Affiliations:** 1 Institute for Advanced Medical Sciences, Hyogo Medical University, 1-1 Mukogawacho, Nishinomiya, Hyogo 663-8501 Japan; 2 Graduate School of Agriculture, Kyoto University, Kitashirakawa-Oiwakecho, Sakyo-ku, Kyoto, 606-8502 Japan; 3 School of Platforms, Kyoto University, Yoshida-Honmachi, Sakyo-ku, Kyoto, 606-8501 Japan; 4 Research Fellow, Japan Society for the Promotion of Science, 5-3-1 Kouji-Machi, Chiyoda-ku, Tokyo, 102-0083 Japan; 5 Graduate School of Agricultural Science, Tohoku University, 468-1 Aoba-ku, Sendai, 980-0845 Japan; 6 International Center for Synchrotron Radiation Innovation Smart, Tohoku University, 468-1 Aoba-ku, Sendai, 980-0845 Japan

**Keywords:** Infectious-disease diagnostics, Electrical and electronic engineering, Assay systems, Sensors and probes

## Abstract

Multidrug-resistant tuberculosis (MDR-TB) is a major clinical problem. Because *Mycobacterium*, the causative agent of tuberculosis, are slow-growing bacteria, it takes 6–8 weeks to complete drug susceptibility testing, and this delay contributes to the development of MDR-TB. Real-time drug resistance monitoring technology would be effective for suppressing the development of MDR-TB. In the electromagnetic frequency from GHz to THz regions, the spectrum of the dielectric response of biological samples has a high dielectric constant owing to the relaxation of the orientation of the overwhelmingly contained water molecule network. By measuring the change in dielectric constant in this frequency band in a micro-liquid culture of *Mycobacterium*, the growth ability can be detected from the quantitative fluctuation of bulk water. The 65-GHz near-field sensor array enables a real-time assessment of the drug susceptibility and growth ability of *Mycobacterium bovis* (BCG). We propose the application of this technology as a potential new method for MDR-TB testing.

## Introduction

The United Nations (UN) and all the World Health Organization (WHO) member states have pledged to adopt the WHO tuberculosis (TB) eradication strategy and the United Nations Sustainable Development Goals (SDGs) with the aim of ending the TB epidemic by 2030^1^. According to the 2021 Global tuberculosis report, approximately 1.3 million people worldwide died of TB in 2020, which is an increase of around 8% compared with 2019^2,3^. However, the global trend in case notification for patients newly diagnosed with TB showed fell 18% from 2019 to 2020. This contradiction may be caused by a reduction in access to TB diagnosis and treatment during this time owing to the coronavirus 2019 (COVID-19) pandemic. A research breakthrough (e.g., a new diagnostic technology, new drugs or a new vaccine) will be required to achieve the goal of ending TB.

The standard first-line treatment for TB is combined antibiotic chemotherapy with isoniazid (INH), rifampicin (RFP), pyrazinamide (PZA), and ethambutol (EB), administered for at least 6 months. However, TB chemotherapy is known to cause the accumulation of genetic abnormalities, regardless of whether the infection is latent or active^4^. Accumulation of genetic abnormalities and incomplete treatment are the main causes of drug-resistant TB (DR-TB). WHO uses five categories to classify cases of DR-TB: INH-resistant TB, RFP-resistant TB (RR-TB), multidrug-resistant TB (MDR-TB), pre-extensively drug-resistant TB (pre-XDR-TB), and XDR-TB. MDR-TB is resistant to both INH and RFP, pre-XDR-TB is resistant to RFP and any fluoroquinolone (a class of secondary anti-TB drugs), and XDR-TB is resistant to RFP, fluoroquinolone, and bedaquiline (BDQ) and/or linezolid. Treating DR-TB requires a course of second-line treatment for 9–20 months^5^. The administration of drugs to which the pathogen is insensitive to patients with DR-TB facilitates the development of resistance to multiple drugs^6^. Currently, 470,000 people worldwide suffer from MDR-TB or RR-TB annually; less than a quarter of such patients begin treatment, and the reported treatment success rates for these patients were only 50% in 2012 and 59% in 2018^3^. Over the last 10 years, shorter regimens of high-dose BDQ and delamanid (DLM) have been used to treat MDR-TB^7–10^. However, the available data from a phase 3 randomized trial to confirm the efficacy of anti-TB drugs are not yet sufficient^11^. Rapid and comprehensive DST technology is essential for the development of effective TB treatment regimens, but current DSTs are complex and inadequate. Over the last two decades, the Middlebrook 7H9 liquid medium-based Bactec MGIT960^®^ culture system has become widely used for rapid DST because of its high sensitivity^12,13^. RFP resistance testing by GeneXpert^®^ is even faster as a rapid molecular testing^14^. However, bacterial culture in solid medium still has been the standard tool for drug-susceptibility testing (DST) since 1903, owing to its high specificity and low cost. The growth cycle of *Mycobacterium*, the causative pathogen of TB, is approximately 16–24 h, even in the proliferative phase; therefore, DST requires 70 days in solid medium and 27 days in Bactec MGIT960^®^^12,13^. To prevent the spread of DR-TB with effective treatment, it is important to monitor susceptibility to anti-TB drugs, both before and during TB treatment. We believe that the rapid measurement of the effects of anti-TB drugs on DR-TB using new technologies can contribute to the selection of appropriate treatments and the management of DR-TB.

In recent years, remarkable progress has been made with high-frequency semiconductor technology, called 5G and Beyond 5G, which plays a central role in next-generation communications^15^. This electromagnetic frequency is higher than microwaves and is in the region called GHz or THz. Because this technology is easier to miniaturize compared with microwaves, it can be used to create small sensor devices. The high frequency sensor technology offers a more compact sensing approach which has the potential in the future for biosensing systems^16,17^. In the spectrum of the dielectric response of biological samples from the microwave to the THz band, a high dielectric constant exists as a consequence of the relaxation of the orientation of the water molecule network, which is overwhelmingly contained. Therefore, in liquid medium measurements, the number of bulk water molecules that is eliminated by small polar molecules, such as proteins and lipids, or that is reduced by hydration is reflected by changes in dielectric constant^18^. Focusing on the imaginary part of the dielectric constant, it is known that the peak of orientation relaxation of the water molecule network appears near 20 GHz, and a gentle slope is drawn from there toward the THz band^19^. In this range, the numerical information regarding bulk water becomes dominant. We previously designed a resonance circuit that oscillates at 60 GHz developed a contact-type sensor that utilizes the resonance frequency shifts according to the dielectric constant of the sensor surface^20^. The electric field exudes from the surface of this sensor to a thickness of approximately 15 µm in the water and the dielectric constant of the space is incorporated into the oscillation frequency of the resonance frequency. Therefore, when the number of water molecules in free water in the culture solution is replaced by solutes or bacteria in this proximity region, the resonance frequency changes, and the amount of change can be measured in near real-time. Furthermore, the sensor has 1488 sensor elements in a 3-mm square that can be driven individually to capture changes in the dielectric constant of each address. Therefore, it is possible to visualize the numerical changes around viable bacteria.

This sensor system can quickly determine the growth of *Escherichia coli* and the action of streptomycin (SM)^21^. This system was able to recognize cell death with higher sensitivity than the fluorescence imaging method by measuring the dielectric constant of HeLa cells in culture over time^22^. Therefore, we tried to measure the change in growth due to the administration of anti-TB drugs (twelve agents) and Ciprofloxacin using *Mycobacterium*, which is a slow-growing bacterium. The advantage of this sensor array is that real-time measurements can capture large amounts of water fluctuations in a small space, allowing the user to distinguish between live/proliferative and dead/dormant bacteria. We describe this as growth ability. If antibiotics reduce the growth ability of bacteria, their effects can be visualized with this system to quickly determine antibacterial activity. This technique is particularly effective because patient sputum contains a mixture of live and dead bacteria, and *Mycobacterium* bacteria grow slowly in culture. The present study used *Mycobacterium bovis* Bacillus Calmette–Guérin (BCG) as a model for TB to investigate the potential use of a sensor array for rapid DST with anti-TB drugs.

## Results

### 65-GHz near-field sensor array and sealing the culture with fluorocarbon

**Figure 1:**
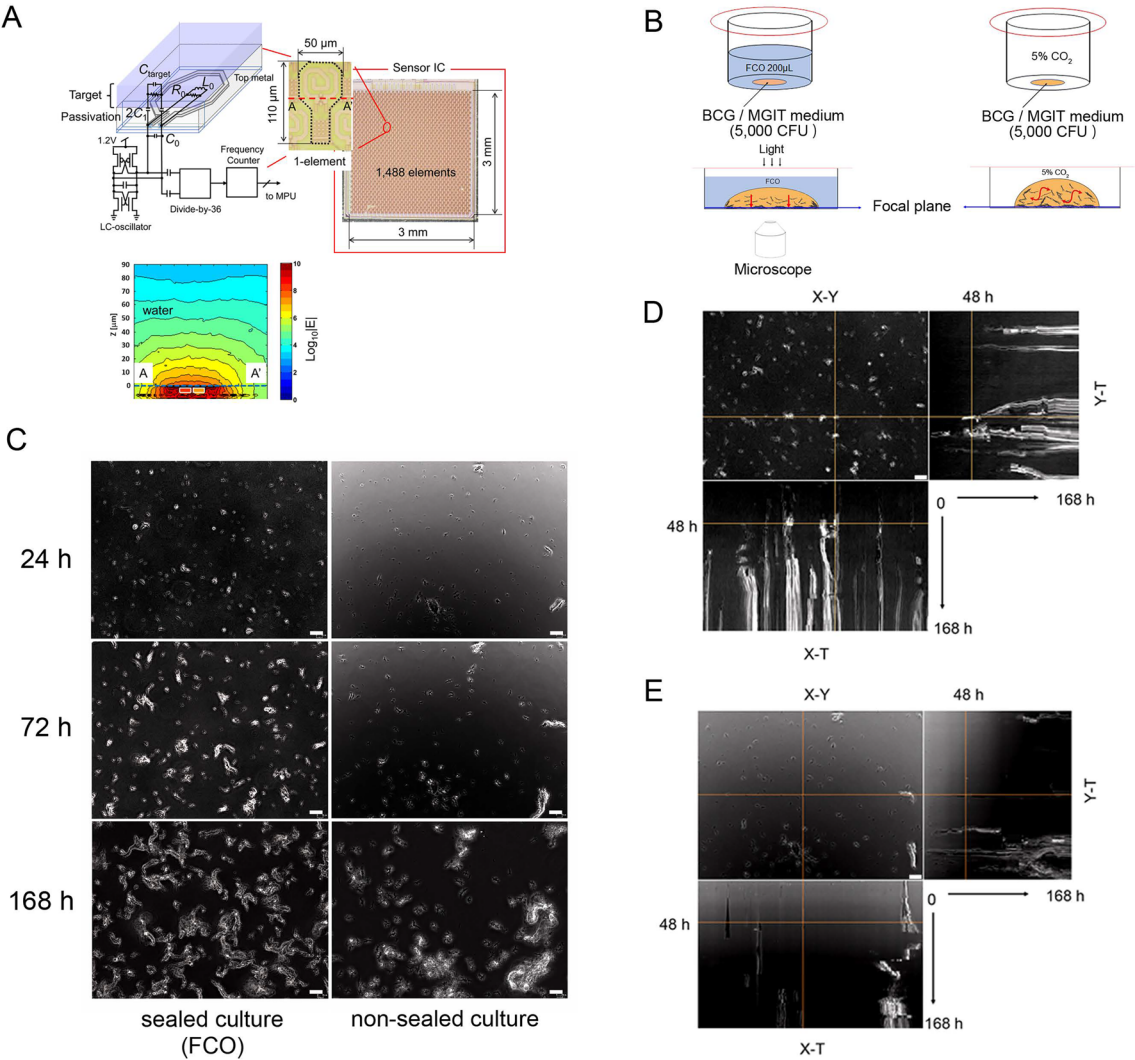
65-GHz sensor and fluorocarbon-sealed culture. (**A**) Schematic of the near-field sensor array. The sensor consists of 1488 elements with a free-running LC oscillator, an injection-locked frequency divider, and a counter circuit. The electric field is located within several dozen micrometers of the sensor surface. The penetration depth of the electric field extends 15 µm from the passivation layer into the water. *The original data for the electric field was modified from Supplemental data in reference^21^. (**B**) Schematic of the culture sealed with oxygen-saturated fluorocarbon (FCO, left) and the non-sealed culture (right). (**C**) Time-lapse microscopy was performed for 168 h with images acquired at 10-min intervals using a transmission inverted microscope. Same-field images of a time-lapse microscope (taken at 24, 72, and 168 h). BCG was stable at the same position in the sealed culture (left lane). There was little formation of BCG bulky mass (right lane). Scale bar = 20 µm. (**D**) Overlaid time-lapse microscopy images of FCO-sealed cultures. A total of 1008 images (acquired at 10-min intervals, 0–168 h) of a single field of view (353 × 256 µm, magnification 20 ×) were overlaid. The cross-section (yellow line) of the XY image is displayed as a slice view of the XT or YT coordinates. BCG growth can be seen as continuous white lines because there was reduced media fluidity (X–T/Y–T views). (**E**) Overlaid time-lapse microscopy images of non-sealed cultures. BCG adhered together by convection in the liquid medium, forming a bulky mass. As the bacterial mass moved, a thick dashed white line was observed over time. Scale bar = 20 µm. CFU, Colony-forming unit; BCG, *Mycobacterium bovis* Bacille Calmette–Guérin; FCO, Oxygen-saturated fluorocarbon.

The resonance frequency of this LC oscillator changes according to the dielectric constant of the sensor surface (mainly on the passivation layer). Figure 1A shows the configuration of the sensor and the electric field distribution on the sensor surface as calculated by electromagnetic field simulation. The result indicates that this sensor is sensitive to the dielectric constant in the region of the bottom surface depth of 15 µm in the water dropped on the surface. Therefore, we adopted a strategy of retaining bacteria on the sensor surface. At first, we investigated the use of several inert solvents as a sealing material for reducing convection in the droplets (Fig. 1B). In this study, oxygenated fluorocarbon (FCO) was used to culture BCG, which is an obligate aerobe. Fluorocarbon (FC) is a gas carrier with relatively low cytotoxicity, and it is also useful for the efficient culture of *E*. *coli*^23,24^. FC was previously investigated as a potential artificial blood substitute^25^, and it is currently used as a tissue perfusion cleanser in ophthalmic surgery^26^. Industrially, it is widely used as a refrigerant for cleaning semiconductor substrates and air conditioners. BCG (5000 colony-forming units [CFU]) was cultured in 5 µl of media, which was sealed with 200 µl of oxygen-saturated FC (Fig. 1B, left). For comparison, closed cultures were performed without sealing (Fig. 1B, right). Time-lapse microscopy was performed for 168 h with images acquired at 10-min intervals using a transmission inverted microscope. BCG was stable at the same position, and there was little formation of BCG bulky masses in the sealed cultures (Supplementary Video 1, Fig. 1C, left column), as compared with in the non-sealed cultures (Supplementary Video 2, Fig. 1C, right column). In a single field (353 × 256 µm, 20 × magnification) time-lapse image series, 1008 images (taken at 10-min intervals, 0–168 h) were overlaid. The yellow line in the X–Y image indicates the cross-section as a slice view of X–T or Y–T coordinates (Fig. 1D,E). BCG growth was visible as continuous white lines (Fig. 1D, X–T and Y–T views). In the non-sealed culture (Fig. 1E), the fluidity of the liquid medium allowed the BCG to mix and adhere to each other, forming a bulky mass, and the formation of thick dashed white lines was observed over time. Time-lapse microscopy confirmed that FCO was able to maintain the BCG in a static position and maintained bacterial growth (Fig. 1C, left).

### Sensing bacterial growth and response to drugs

The 65-GHz frequency band used in our sensor is sensitive to changes in the number of bulk water molecules but is largely unaffected by larger molecules and ions^18,21^. When bacteria in the liquid medium settled onto the surface of the sensor, the main factors affecting the difference in resonance frequency (MHz) were: (1) the total bacterial volume, (2) the number of bulk water molecules, and (3) changes in the dielectric constant owing to bacterial metabolites. All of these changes occur as a consequence of the activity of live bacteria. Therefore, “growth ability” was defined as the difference in resonance frequency steadily increasing by + 5 MHz or more within a few hours. After confirming the growth ability, an antibacterial agent was added to the liquid medium, and the BCG susceptibility to that agent was evaluated from the change in resonance frequency (Supplementary Fig. 1A). Each point on the heatmap corresponds to a single element of the sensor array, and the amount of change in resonance frequency in each element is indicated by the difference in frequency (MHz). Growth was measured from 3–24 h and is indicated by blue (difference ≥ + 5 MHz) on the heatmap. When BCG was removed or growth ability was impaired by injection of antibacterial agent, the resonance frequency became nearly the same as it was at the start of culture (yellow). The edges of the culture medium appear red (negative values) on the heatmap, indicating that the volume increased after injection of the drug or medium. When the growth ability was not impaired, the difference (MHz) of the element was indicated as blue in the heatmap. The growth ability was measured in real-time by analyzing the heatmap. However, growth was not analyzed for the FC region, which had no culture medium on the surface of the sensor (light green). Simultaneous measurement was performed with two sensors per incubator (Supplementary Fig. 1B). In cases of no drug sensitivity, there was a large positive difference in the frequency and a large amount of variation (see error bars), as shown in line X of Supplementary Fig. 1C. Drug sensitivity is shown by the lines Y and Z. A histogram analysis was performed after drug administration for each experiment. The distribution on the x-axis indicates bacterial susceptibility to the tested drug according to the change in resonance frequency (MHz) (Supplementary Fig. 1D).

### Heatmap analysis and fluorescence staining of live or dead bacteria

**Figure 2:**
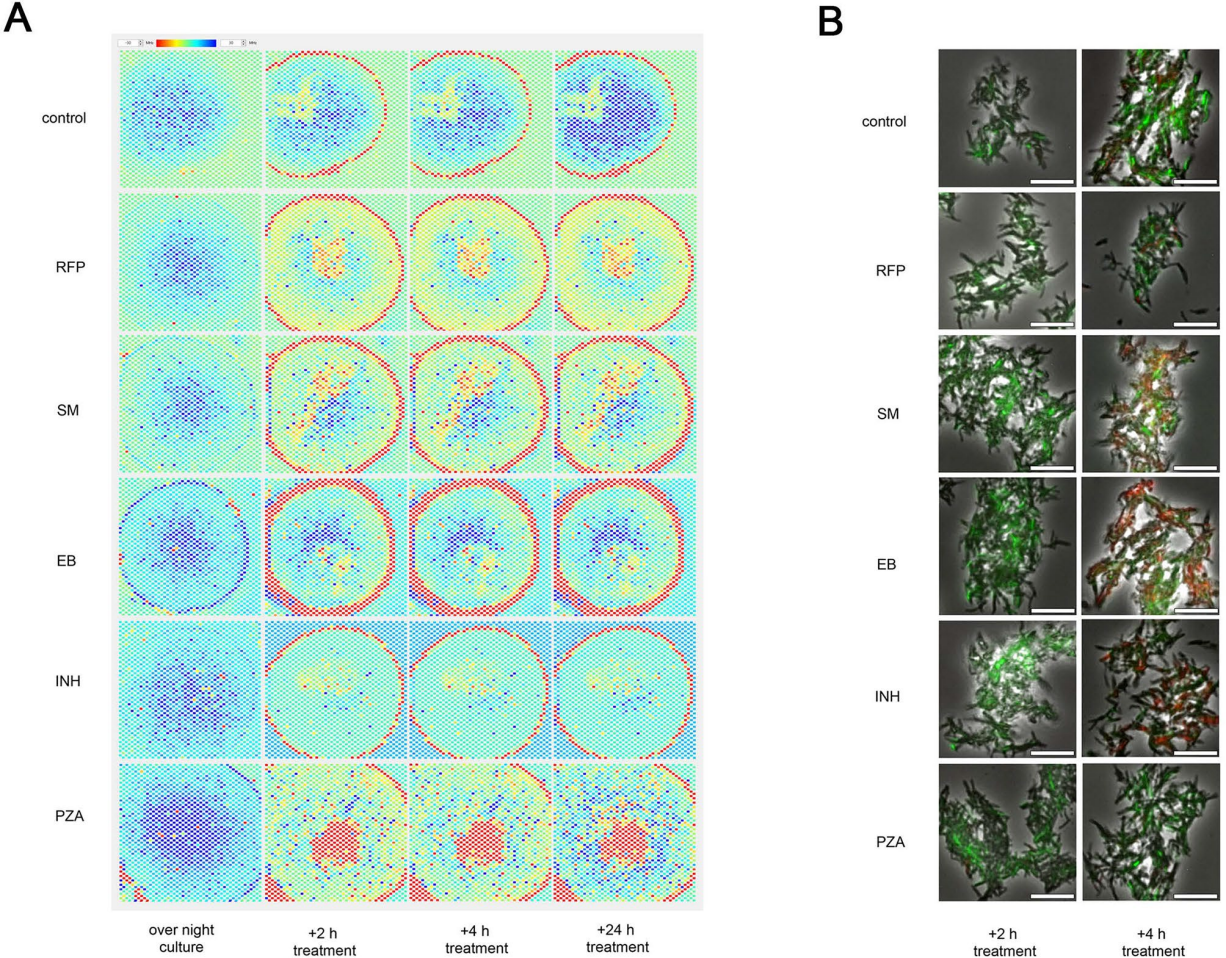
Growth ability and drug response sensing. (**A**) Drug susceptibility of BCG to first-line anti-TB drugs. RFP, EB, INH, PZA, and SM were each administered to a culture of BCG, and the resulting difference in resonance frequency (MHz) over 24 h is shown in heatmaps. (**B**) BCG viability assay. After 2 or 4 h of shaking liquid culture with antibacterial agent, the dead bacteria (red) and live bacteria (green) were stained with live/dead reagent. Scale bar = 20 µm. *RFP* rifampicin, *EB* ethambutol, *INH* isoniazid, *PZA* pyrazinamide, *SM* streptomycin.

After BCG was cultured overnight, anti-TB drugs were injected at 2 × the Centers for Disease Control and Prevention (CDC)-recommended critical drug concentration. The differences in resonance frequency (MHz) are shown in heatmaps (Fig. 2A). From 2–24 h after drug injection, the differences in resonance frequency decreased for cultures injected with RFP, streptomycin (SM), EB, or INH compared with control cultures (injected with MGIT medium), suggesting that these drugs suppressed bacterial growth. However, since BCG is known to be intrinsically PZA-resistant^27^ and PZA is inactive at pH 6.7 (the standard MGIT pH), and the blue areas on the heatmap increased after 24 h. A viability assay (Fig. 2B) was performed to compare the drug sensitivity findings with the dielectric constant detected by the sensor. After the liquid culture in MGIT containing the target antimicrobial with shaking for 2 or 4 h, the BCG culture was stained with live/dead reagent (Fig. 2B). BCG with intact cell membranes are stained with green fluorescence (SYTO^®^9) and cells with damaged membranes are stained with red fluorescence (Propidium iodide). At 4 h after drug administration, dead cells (red) were observed in the cultures exposed to SM, EB (a protein synthesis inhibitor), or INH (a monoamine oxidase inhibitor), but live cells (green) were observed in the cultures exposed to PZA or RFP (a time-dependent, RNA polymerase inhibitor). A comparison of the heatmap analysis and fluorescence staining results following anti-TB drug exposure suggests that the dielectric constant detected by the sensor does indicate changes in the growth ability prior to changes in the bacterial structure, regardless of the pharmacological mechanism of action.

### Comprehensive DST using the sensor

**Figure 3:**
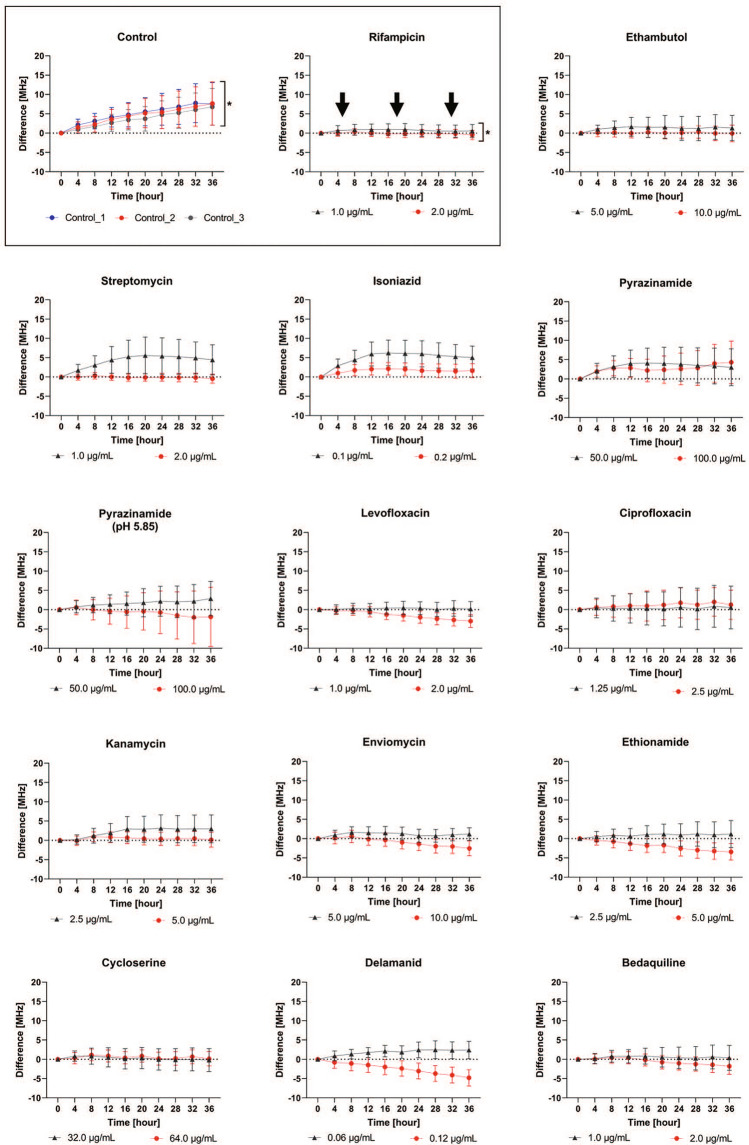
Comprehensive drug susceptibility testing using the sensor. BCG was cultured on the sensor, and then one of two different concentrations of first-line or second-line anti-TB drugs were injected into each culture. The mean difference in resonance frequency of all evaluable elements (MHz) is shown for the cultures treated with the low concentration (black triangle) or high concentration (red circle). The error bars show the standard deviation (SD) of the element measurements. As shown in the upper left rectangle, the mean difference and SD increased over time (*) in the control, but no apparent changes in these factors were observed following RFP administration (↓, *). RFP, EB, SM, INH, LVFX, KM, EVM, TH, CS, DLM, and BDQ each had an antibacterial effect against BCG when used at the high concentration dose. PZA and ciprofloxacin also caused a large SD and did not show any obvious antibacterial activity against BCG. For LVFX, EVM, TH, DLM, and BDQ, the resonance frequency change (MHz) was negative, and the SD was small for the high-concentration dose. The time required for stable drug efficacy was a maximum of 16 h, which is nearly the length of the BCG growth cycle. *SD* standard deviation, *LVFX* levofloxacin, *KM* kanamycin, *EVM* enviomycin, *TH* ethionamide, *CS* cycloserine, *DLM* delamanid, *BDQ* bedaquiline.

To determine the time course and drug concentration required for making diagnoses with this sensor, measurements were taken every 5 min for 36 h after anti-TB drug administration (Fig. 3). BCG (30,000 CFU) was sealed by FCO and cultured on the sensor. After confirming the growth ability, first-line or second-line anti-TB drugs were injected into the culture medium at one of two different concentrations. The mean value of the difference in resonance frequency (MHz) of all evaluable elements is shown for the low-concentration (Fig. 3, black triangle) and high-concentration (Fig. 3, red circle) doses. The error bars indicate the standard deviation (SD). A culture was designated as sensitive to a drug if there was no increase in the difference (MHz) and no increase in the SD after drug administration. As shown in the rectangle in Fig. 3, the mean difference (MHz) and SD increased over time (*) in the controls, but there was no apparent increase after RFP administration (↓, *). RFP, EB, SM, INH, levofloxacin (LVFX), kanamycin (KM), enviomycin (EVM), ethionamide (TH), cycloserine (CS), DLM, and BDQ had an antibacterial effect against BCG at the high concentration. For PZA, the DST was performed both at pH 6.7 (the standard MGIT pH) and at pH 5.85 (the pH recommended by the manufacturer). PZA did not have any obvious antibacterial effect under either set of conditions. These results were expected; BCG is known to be resistant to PZA^27^. The ciprofloxacin test had a large SD and did not show an antibacterial effect. For cultures exposed to LVFX, EVM, TH, DLM, or BDQ, the resonance frequency change was negative, and the SD was small at the high-concentration dose. The measurement time series showed that the time required for stable drug efficacy was a maximum of 16 h, which is nearly equivalent to the growth cycle of BCG.

### Histogram analysis of drug susceptibility at 16 h

A histogram analysis of the cultures was performed using the 16-h data shown in Fig. 3. The x-axis is the distribution of the difference in resonance frequency (MHz) of each element in Supplementary Fig. 2. There were three control experiments, and one experiment for each drug. A distribution located around X = 0 with a large Y-value indicated low growth. In contrast, a broad distribution on the x-axis indicated high growth. Anti-TB DST was performed with a high concentration of antimicrobial (red bars) and a low concentration of antimicrobial (blue bars). The y-axis indicates the fraction (%) of all the measured elements. The bacterial cultures fluctuated upon the injection of drug or medium; therefore, the number of elements evaluated in each assay was different. The mean value of evaluated elements was 1039 (range: 745–1260, SD: 150). BCG was sensitive to RFP, LVFX, CS, DLM, and BDQ at low concentrations. For EB, SM, INH, KM, EVM, and TH, it was speculated that there was a critical breakpoint between the high and low concentrations. PZA and ciprofloxacin did not have any obvious effect at either concentration. For LVFX, EVM, TH, and DLM, most elements showed a negative difference value at the high concentration. Possible reasons for this include an increase in dead bacteria owing to drug efficacy and an increase in the number of bulk water molecules around the bacteria. For most cultures, the effective concentration and antibacterial activity could be determined from the histogram analysis at 16 h after drug administration.

## Discussion

### Characteristics of *Mycobacterium* and usefulness of dielectric constant measurement

*Mycobacterium species* produce mycolic acids, and they form the major component of the cell wall. Mycolic acids make the organism more resistant to chemical damage and dehydration, and limit the effectiveness of hydrophilic antibiotics and biocides. Mycolic acids are also known to protect the bacteria from toxic enzymes and allow them to escape the immune response^28^. For these characteristics, mycolic acids impart *M. tuberculosis* with unique properties that defy medical treatment. In addition, *Mycobacterium* species become dormant or to grow even more slowly than usual under acidic conditions, which allows these bacteria to survive for a long time when using immune cells, such as in macrophages, or phagolysosomes as a host^29,30^. Because *Mycobacterium* species have no mobility and strong adhesiveness due to mycolic acids, their typical growth mode is “cording”, i.e., they grow in a filamentous form^31^. Consequently, a quantitative growth evaluation of these bacteria based on turbidity is technically difficult. These features make rapid culture testing of *Mycobacterium* species difficult and limit the drug susceptibility of these bacteria. However, about 70% of the components of *Mycobacterium* are water, and a large amount of bulk water is required for their growth. In the 65-GHz band, quantitative changes in bulk water dominate the dielectric constant. Therefore, this sensor can measure drug sensitivity in real-time from the permittivity around the bacteria after administration of antibacterial agents.

### The treatment regimen for TB and current diagnostic tools for rapid DST

Monotherapy with a first-line therapeutic agent is contraindicated for TB because the causative bacteria grow slowly and are consequently less sensitive to antibiotics and develop mutation-induced resistance. The standard treatment regimen for initial TB treatment is combination chemotherapy with four drugs (RFP, INH, PZA, EB) for 6 months or longer (Supplementary Fig. 3). Most first-line treatment failures are due to treatment discontinuation, inadequate regimen, or the acquisition of new drug resistance during long-term combination chemotherapy. Essentially, during long-term antibiotic treatment, it is necessary to monitor changes in the growth ability and drug susceptibility, as well as to check for the negative conversion of sputum cultures. Current clinical DST methods for DR-TB include solid medium culture, liquid culture (BACTEC MGIT960^®^)^32^, resistance gene detection by gene analysis (Line probe assay/LiPA)^33^, nucleic acid amplification testing (Xpert MTB/RIF)^14^, and the whole-genome analysis method^34,35^. Xpert MTB/RIF is a powerful diagnostic tool for RFP resistance and its use is improving the diagnosis of DR-TB. In 2020, 71% (2.1/3.0 million) of cases of DR-TB were diagnosed with RFP resistance worldwide. This percentage has increased over the past couple years, from 50% (1.7/3.4 million) in 2018 and 61% (2.2/3.6 million) in 2019. There has also been an attempt to predict bacterial drug susceptibility via direct sputum whole-genome sequencing^34^. Because gene analysis indirectly diagnoses drug resistance by detecting a mutation in a specific gene region, it is difficult to apply this method to directly and comprehensively diagnosing the susceptibility of bacteria to various drugs. For this reason, a new quantitative phenotypic drug susceptibility test assay for a panel of 14 anti-TB drugs has been developed^36^. This is a method in which *Mycobacterium* bacteria are cultured on a 96-well dry plate for 14 days, after which the mean inhibitory concentration is measured by microscopic observation. However, with these diagnostic methods, it is practically difficult to rapidly diagnose drug susceptibility at low cost using a large number of patient-derived bacterial samples. Additionally, because of the large number of patients with TB and limited diagnostic resources, it is not appropriate to use gene analysis, nucleic acid amplification, or whole genome analysis as standard DST methods.

### The clinical value of sensor array technology in the treatment for TB

The treatment success rate for MDR/RR-TB improved in 2018 (59%) owing to the optimization of therapeutic agents^3^. Consequently, the WHO DR-TB expert meeting proposed the use of longer MDR-TB regimen and shorter regimen options that combine 17 types of anti-TB drugs into three groups^11^. However, the effective use of these therapies will require the development of research innovations in DST. The development of a new sensing technology may allow for the selection of appropriate TB drug candidates, as well as the easy identification of viable bacteria, which could lead to a new treatment regimen. In our data, the histogram of resonance frequency following drug administration shows various patterns, which may reflect differences in drug pharmacokinetics; therefore, this sensing technology may be useful for drug discovery screening and pharmacological tests. Near-fields sensor array with CMOS oscillators will enable the development of a rapid and comprehensive DST method that is suitable for real-time evaluation of the growth ability and drug resistance of slow-growing bacteria.

### The possible future applications of sensor array technology

Unlike the PCR methods, there is no need to target each gene for testing. This means that the sensor method is not specific for TB diagnosis, but because it is a phenotypic susceptibility test, drug susceptibility to all anti-TB drugs, including INH, can be obtained at the initial treatment stage. It is expected to significantly reduce the time required to determine results compared to DST using the culture methods (Supplementary Fig. 4). In addition, sensor-based bacteriologically monitoring of medication effects enables early detection of the emergence of drug-resistant TB during treatment, enabling immediate response to drug-resistant bacteria. Early detection of the emergence of drug-resistant bacteria during treatment and prompt decision to switch to second-line anti-tuberculosis drugs might lead to the prevention of the emergence of multidrug-resistant tuberculosis bacteria and the optimization of treatment (Supplementary Fig. 3).

## Materials and methods

### Bacterial strain, culture medium, and chemicals

*Mycobacterium bovis* (BCG, Tokyo strain) was purchased from Japan BCG Laboratory (Tokyo, Japan) and maintained in BD BACTEC™ MGIT™ medium supplemented with OADC Enrichment and PANTA antibiotic mixture (Becton Dickinson Co., 245122, Franklin Lakes, NJ) at 37 °C. Mid-log-phase BCG was prepared at concentrations of 1000 CFU/µl for time-lapse microscopy and 10,000 CFU/µl for drug susceptibility testing in MGIT media by using a bacterium counting chamber (A161, SANSYO, Tokyo, Japan). RFP, INH, PZA, SM, EB, LVFX, TH, and CS were obtained from FUJIFILM Wako (Tokyo, Japan). KM and EVM were obtained from Nacalai Tesque (Tokyo, Japan) and Asahi-Kasei Pharma (Tokyo, Japan), respectively. DLM and BDQ were obtained from Selleck (Houston, TX). These chemicals were dissolved in deionized water (INH, PZA, SM, EB, TH, KM, EVM, and CS), methanol (RFP, TH), 0.1 N hydrochloride (LVFX), or dimethyl sulfoxide (DLM, BDQ), and then sterilized using 0.22-µm MILLEX-GV syringe filters (Millipore, Burlington, MA) prior to being added to MGIT media under aseptic conditions. Perfluorocarbon (Fluorinert™, FC-3283) was obtained from 3M (Maplewood, MN). Live/Dead™ BacLight™ bacterial viability reagent was purchased from Thermo Fisher Scientific (L13152, Waltham, MA). Detailed information on the antibacterial agents used in this study is shown in Supplementary Table 1.

### Sealed culture and time-lapse microscopy

The bacterial suspension (5000 CFU of BCG in 5 µl of medium) was placed into a single-well glass dish (IWAKI, 3971-101, Tokyo, Japan) and sealed with FCO. The culture well was then covered with glass and sealed with a liquid pressure-sensitive adhesive (BBX909, Cemedine™, Tokyo, Japan). Time-lapse microscopy images (BioStation IM^®^, Nikon, Tokyo, Japan) were taken every 10 min for 168 h at 37 °C. The original magnification was 20 ×. Microscopic images were constructed using BioStation IM software (version 2.30, Nikon). Slice view images were constructed using NIS-Elements (version 5.10.00, Nikon).

### Liquid culture and bacterial viability assay

BCG was cultured with shaking in a BD BBL™ MGIT™ mycobacteria growth indicator tube (4 ml, 37 °C, 300 rpm). The increase in CO_2_ concentration due to bacterial growth was confirmed with a fluorescence indicator. After a pellet of BCG cells was collected via centrifugation at 3000 rpm for 10 min, the cell concentration was adjusted to 10,000 CFU/µl. RFP, SM, EB, INH, and PZA were added to 500 µl (5,000,000 CFU) of BCG culture at 2 × the CDC-recommended critical concentration, and the culture was then placed in an incubator-shaker (37 °C, 300 rpm). After 2 or 4 h, the BCG cultures were collected, stained with Live/Dead BacLight bacterial viability reagent in accordance with the manufacturer’s protocol, and subjected to fluorescence microscopy (FITC/Texas red) on a Nikon BioStation.

### 65-GHz near-field sensor array and culture system

The sensor array consisted of 1488 LC oscillators made via Complementary metal–oxide–semiconductor (CMOS) technology in a 3-mm square, and its oscillation frequency was designed to be 65 GHz. Bulk water formed a hydrogen bond network with a disappearance time on the order of picoseconds. The theoretical peak of the dielectric constant in the original water molecules was approximately 20 GHz based on the rotational relaxation according to dielectric spectroscopy data. Because the 65-GHz oscillation frequency of this sensor was located on the slope of its large peak, it was sensitive to changes in the amount of bulk water. The surface of the oscillator was covered by SiO_2_ passivation, and the surface was sensed by contacting the sample. Each oscillator was 50 µm × 110 µm in size (including the inductor), and the electric field was localized in the proximity region approximately 15 µm from the passivation layer. Because the resonance frequency of the oscillator (described below) included dielectric constant in the vicinity of the oscillator, the resonance frequency *f*_res_ changed according to the change in dielectric constant (${\varepsilon }^{*}={\varepsilon}_{0} ({\varepsilon}_{r}-j{\varepsilon}_{i})$) of the sample.

1 $$\begin{aligned} {f}_{\mathrm{res}}&=\frac{1}{2\pi \sqrt{{L}_{0}{C}_{\text{eff}}}} \\ {C}_{\text{eff}}&={C}_{0}+{{C}_{1}C}_{\mathrm{air}}\frac{{{\varepsilon }_{r}C}_{1}+{C}_{\mathrm{air}}({\varepsilon }_{r}^{2}+{\varepsilon }_{i}^{2})}{{({C}_{1}+{\varepsilon }_{r}{C}_{\mathrm{air}})}^{2}+{({\varepsilon }_{i}{C}_{\mathrm{air}})}^{2}} \end{aligned}$$

where ${C}_{\text{eff}}$, ${L}_{0}$, ${C}_{0}$, ${C}_{1},$ and ${C}_{\mathrm{air}}$ are, respectively, the effective capacitance (including the passivation layer on the sensor surface), the inductance, the circuit capacitance, the capacitance of the passivation layer, and the capacitance with vacuum permittivity (8.85 pF/m) calculated by electromagnetic field simulation.

When one oscillator captured data with a gate time of 200 µs, the time to collect data for all 1488 elements was 0.5 s or less. The frequency resolution of each oscillator under these conditions was estimated to be 0.33 MHz. Because the dielectric constant of water highly depends on the temperature in the 65-GHz band, temperature sensors were mounted at the four corners of the sensor; when the 1488 data points were captured, the temperature at that time was also recorded. The temperature change and the change in resonance frequency using ultrapure water were measured at − 20 MHz per + 1 °C. Therefore, to control the housing of the sensor, an air circulation incubator (Cool Incubator ICI-1, ASONE Co., Ltd., Osaka, Japan), a Peltier temperature controller (VPE-35, VICS Co., Ltd., Musashino, Japan), and a panel glass heater (ST-H070, BLAST Co., Ltd., Kawasaki, Japan) were used. The temperatures were set to 36 °C, 40 °C, and 42 °C for the air incubator, Peltier controller, and panel glass heater, respectively, and the temperature was controlled such that the thermometer with a built-in sensor would reach 36 °C. The culture system of the sensor is shown in Supplementary Fig. 1. The measured data were saved to a computer via a USB cable.

### Sealed culture on sensor and drug susceptibility testing

BCG (30,000 CFU/3 µl medium) was placed on the sensor. FCO (200 µl) and MGIT medium (100 µl) were then layered, and an adhesive film (Ultra Amp plate seal 36590, Sorenson BioScience, Inc., Salt Lake City, UT) was used to ensure an air-tight seal. A layer of MGIT medium was used to avoid water evaporating from the bacterial suspension (Supplementary Fig. 1). BCG was cultured at 37 °C for 3 h. The difference in the resonance frequency (MHz) was measured at 5-min intervals for up to 36 h. The BCG growth ability was confirmed if the difference increased by + 5 MHz or greater during culturing. Next, an antibacterial drug or MGIT medium (2 µl) was injected into the bacterial suspension. The final concentration of each antibacterial drug is listed in Supplementary Table 1. For the first-line therapeutic agents (RFP, INH, EB, and PZA) and SM, DST was attempted using drug concentrations of 2 × the CDC-recommended critical concentration in the MGIT liquid culture. For the other tested drugs, susceptibility testing was conducted at two different drug concentrations (shown in Supplementary Table 1). Each drug was diluted in BD BACTEC MGIT medium (pH 6.74) in accordance with the manufacturer’s instructions. Because the manufacturer of the PZA drug susceptibility test recommended that it be performed at pH 5.85, this test was performed at pH 5.85 as well as at the pH 6.74 used testing the other drugs. For drug-free controls, an equal volume (2 µl) of MGIT liquid medium was administered instead of an antibacterial drug.

### Measurements and data analysis

The magnitude of change in resonance frequency of an element is indicated by the average difference in frequency (MHz) and SD (shown in figures as the error bar). From the start of culture to the end of measurement, the entire region where the bacterial suspension was present on the element and for which no change of − 10 MHz or less was observed before or after injection of the drug solution was used as the measurement area. Of the 1488 elements on the sensor array, 745–1260 elements (average: 1039; SD: 150) were automatically selected by the computer program for use in analysis. In accordance with these criteria, sensor elements that lost bacteria from the near-field after the injection of antibacterial drug or liquid medium were removed by an automated program. The growth ability was indicated by the mean difference in resonance frequency (MHz) starting from 0 h and the SD. DST was performed at two concentrations for some drugs (Supplementary Table 1). There were three control experiments, and one experiment for each drug. Because the temperature was controlled within ± 0.1 °C, the effect of each drug on BCG was determined from the time course of the difference (MHz) of each sensor element without correcting for changes in temperature. A histogram analysis was performed at 16 h after drug administration by using elemental measurements from each sensor (Fig. 3). The data analysis and graphs were prepared by using GraphPad Prism™ (version 9.2.0, GraphPad Software, Inc., San Diego, CA).

## Supplementary Information

Supplementary Video 1.

Supplementary Video 2.

Supplementary Legends.

Supplementary Figure 1.

Supplementary Figure 2.

Supplementary Figure 3.

Supplementary Figure 4.

Supplementary Table 1.

## Data Availability

The datasets used and/or analyzed during the current study are available from the corresponding author on reasonable request.
