# Supplementary material for: Near-field sensor array with 65-GHz CMOS oscillators can rapidly and comprehensively evaluate drug susceptibility of Mycobacterium
Source: Sci Rep. 2023 Mar 7;13:3825. doi: 10.1038/s41598-023-30873-9 (PMC9990582; doi:10.1038/s41598-023-30873-9)
Supplement: Supplementary file 3 — Supplementary Legends. [file 41598_2023_30873_MOESM3_ESM.docx]

**Supplementary Figure legends, Table, Videos**

**Supplementary Figure 1. Anti-TB drug administration and growth analysis.**

**(A)** Schematic of the sealed culture and drug susceptibility testing method. The medium on the top prevented the evaporation of bulk water from the culture. Growth was measured in real-time using heatmaps showing the change in resonance frequency.

**(B)** Bacterial growth experiment in a temperature-controlled environment. A cloning ring was placed on the sensor to form a culture well. Simultaneous measurement was performed with two sensors per incubator. The measurement data were output to a computer via a controller and USB port.

**(C)** Each point on the heatmap corresponds to one element of the sensor array, and the change in the resonance frequency for each element is indicated as the difference (MHz). The mean difference and standard deviation (error bars) of all evaluable elements are indicated. X, MGIT medium; Y, Rifampicin; Z, Delamanid.

**(D)** Histogram analysis after drug administration. The distribution on the x-axis indicates drug susceptibility according to the change in resonance frequency (MHz).

**Supplementary Figure 2. Histogram analysis of drug susceptibility at 16 h.**

Drug susceptibility testing for BCG with anti-TB drugs was performed at a high-concentration (red) and low-concentration (blue) doses. Change in the frequency data for 16 h after drug administration is shown in the histograms. The x-axis indicates the relative resonance frequency change (MHz), and the y-axis indicates the fraction (%) of all of the measured elements. There were three experimental controls, and one experiment for each of the tested drugs. BCG was sensitive to RFP, EB, SM, INH, LVFX, KM, EVM, TH, CS, DLM, and BDQ. Antibacterial effects were observed at high concentrations of EB, SM, INH, KM, EVM, and TH, and it was speculated that there was a critical breakpoint between the two concentrations. PZA and ciprofloxacin did not have sufficient efficacy. The effective concentration and antibacterial activity of each drug were determined from the histogram analysis at 16 h.

**Supplementary Figure 3 The monitoring of viable TB and a decision to switch to second-line anti-TB drugs.**

Standard tuberculosis treatment protocols may delay second-line treatment due to the lack of surveillance information on viable tuberculosis. Periodic drug susceptibility monitoring may allow a rapid decision to switch to second-line anti-TB drugs, even if resistance emerges during treatment.

**Supplementary Figure 4. The phenotypic drug susceptibility testing within 24 hours.**

Tuberculosis diagnosis flow is optimized by introducing the sensor method. The left is the current diagnosis flow, using culture methods. The right is the flow with the sensor methods. The sensor-based phenotypic drug susceptibility testing results in a rapid determination of the initial treatment drug, and it reduces the time for the diagnosis.

**Supplementary Table 1. Antibacterial agents recommended for use against Mycobacterium tuberculosis.**

Details of the first-line and second-line anti-tuberculosis drugs used in this study are shown. The effective concentration of an anti-tuberculosis drug is controversial and depends on the strain, medium, culture method, and drug resistance. * indicates the recommended critical drug concentration in MGIT liquid culture according to the CDC Standard Strain Panel. The solvent recommended by the manufacturer was used to dissolve each drug. MGIT medium was used to make the final adjustment to obtain the two tested concentrations. The measured pH of MGIT medium was 6.7; only PZA was tested at both pH 5.8 and 6.7.

**Supplementary Video 1. Time-lapse microscopy of a BCG culture sealed with fluorocarbon.**

Time-lapse microscopy was performed for 168 h with images acquired at 10-min intervals. BCG bacteria were stable on the surface of the culture dish, and there was little formation of bulky mass. Video shown at 100 frames per second; scale bar = 10 µm.

**Supplementary Video 2. Time-lapse microscopy of a BCG culture that was not sealed with fluorocarbon.**

BCG bacteria flowed in the liquid medium and adhered to each other, resulting in the formation of bulky mass. Video shown at 100 frames per second; scale bar = 10 µm.
