# Supplementary figures and images for: Near-field sensor array with 65-GHz CMOS oscillators can rapidly and comprehensively evaluate drug susceptibility of Mycobacterium
Source: Sci Rep. 2023 Mar 7;13:3825. doi: 10.1038/s41598-023-30873-9 (PMC9990582; doi:10.1038/s41598-023-30873-9)

**A**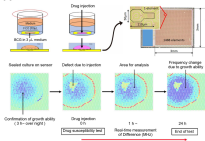**B**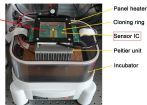**C**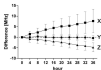**D**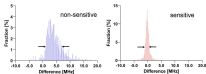

Supplement: Supplementary file 4 — Supplementary Figure 1. [file 41598_2023_30873_MOESM4_ESM.pdf]

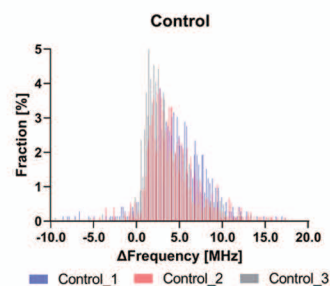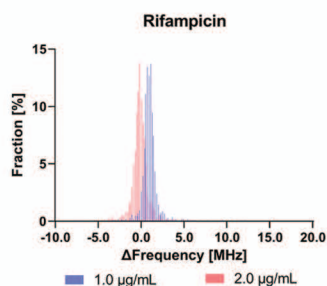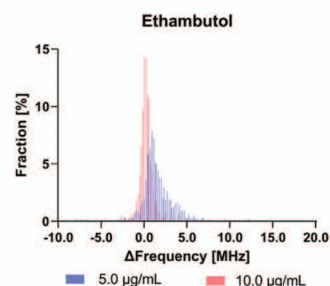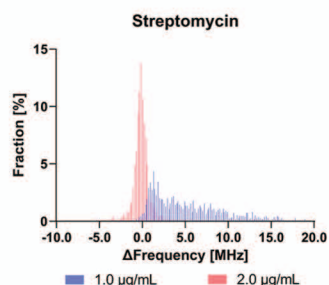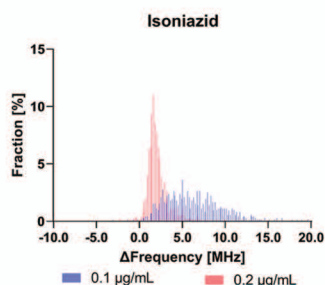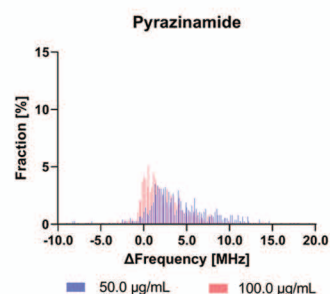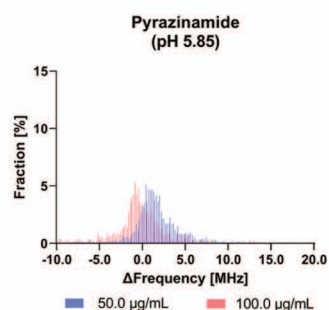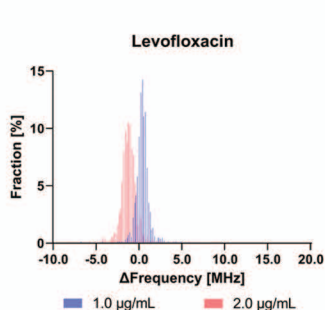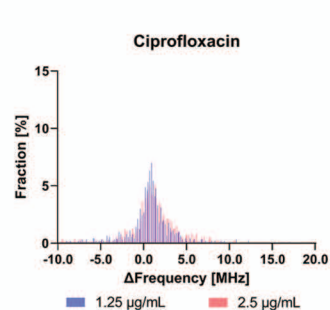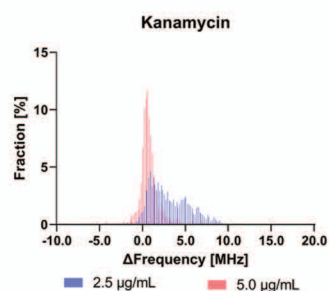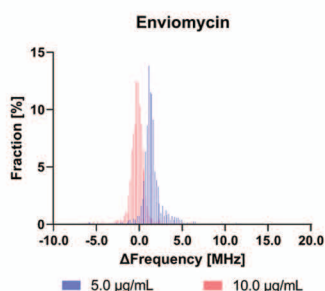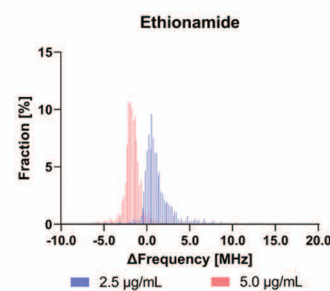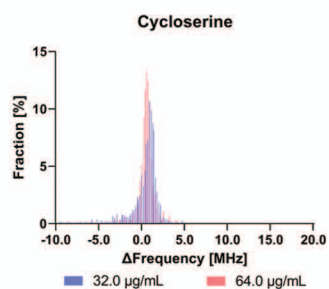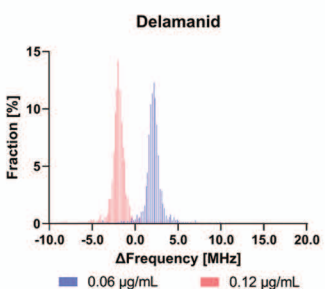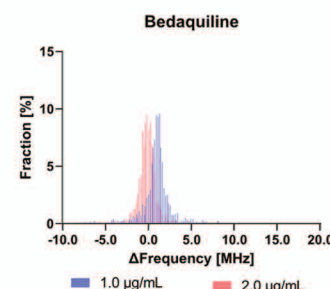

Supplement: Supplementary file 5 — Supplementary Figure 2. [file 41598_2023_30873_MOESM5_ESM.pdf]
