# Supplementary material for: Near-field sensor array with 65-GHz CMOS oscillators can rapidly and comprehensively evaluate drug susceptibility of Mycobacterium
Source: Sci Rep. 2023 Mar 7;13:3825. doi: 10.1038/s41598-023-30873-9 (PMC9990582; doi:10.1038/s41598-023-30873-9)
Supplement: Supplementary file 6 — Supplementary Figure 3. [file 41598_2023_30873_MOESM6_ESM.pdf]

Standard treatment protocol for TB and monitoring using sensor method

Monitoring by clinical findings like blood tests

- Lack of timely information to make decisions about changing anti-TB drugs delays the timing of switching to second-line drugs

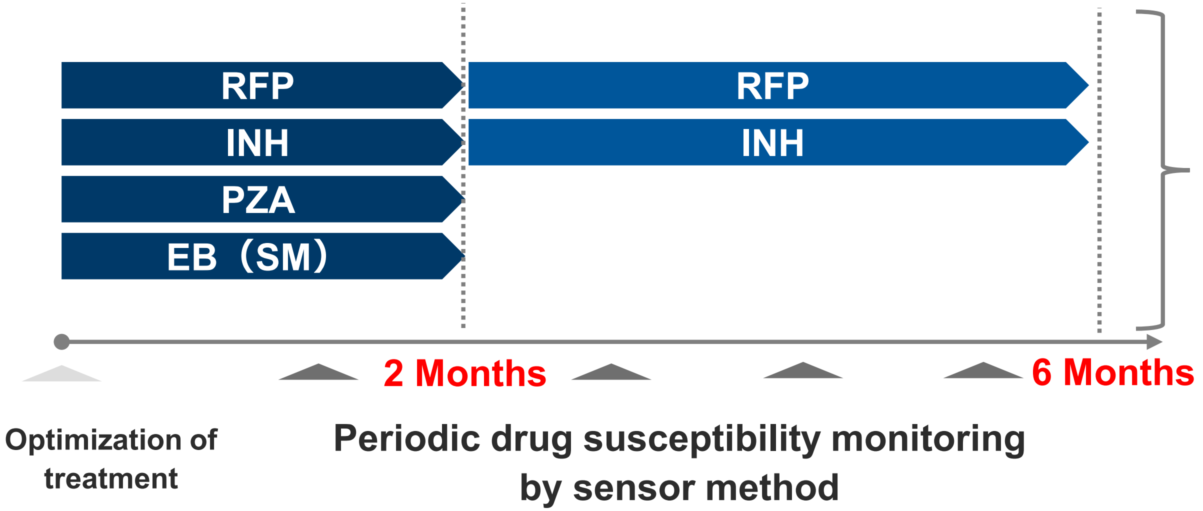

Bacteriological efficacy monitoring using sensor method

☑ Rapidly detect the emergence of resistant bacteria during treatment, allowing prompt decision to switch to second-line anti-tuberculosis drugs

Change drugs promptly according to the principle of use of anti-TB drugs when the emergence of resistant bacteria is detected

Category	Characteristic	Drugs
First-line drug (a)	The most powerful anti TB action	Rifampicin（Rifabutin）
		Isoniazid
		Pyrazinamide
First-line drug (b)	effective in combination with first line drugs (a)	streptomycin
		Ethambutol
Second-line drug	expected to be effective in combination with multiple drugs	Levofloxacin
		Kanamycin
		Ethionamide
		Enviomycin
		para-aminosalicylic acid
		Cycloserine
		Linezolid
		Clofazimine
Multi-drug resistant tuberculosis drugs	Multidrug-resistant TB only	Delamanid
		Bedaquiline
