# Supplementary material for: Near-field sensor array with 65-GHz CMOS oscillators can rapidly and comprehensively evaluate drug susceptibility of Mycobacterium
Source: Sci Rep. 2023 Mar 7;13:3825. doi: 10.1038/s41598-023-30873-9 (PMC9990582; doi:10.1038/s41598-023-30873-9)
Supplement: Supplementary file 7 — Supplementary Figure 4. [file 41598_2023_30873_MOESM7_ESM.pdf]

TB diagnosis flow may be optimized as follows by introducing the sensor method.

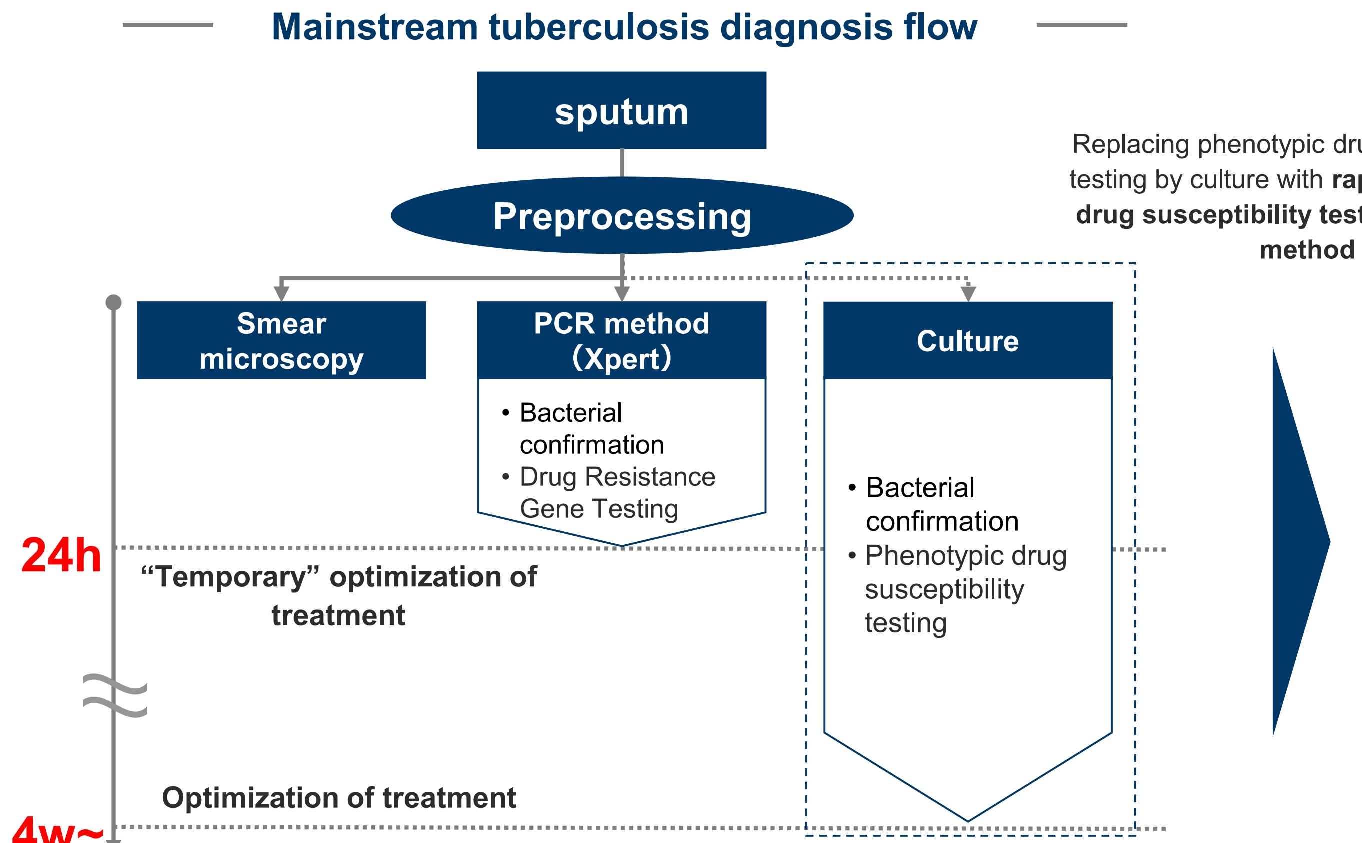

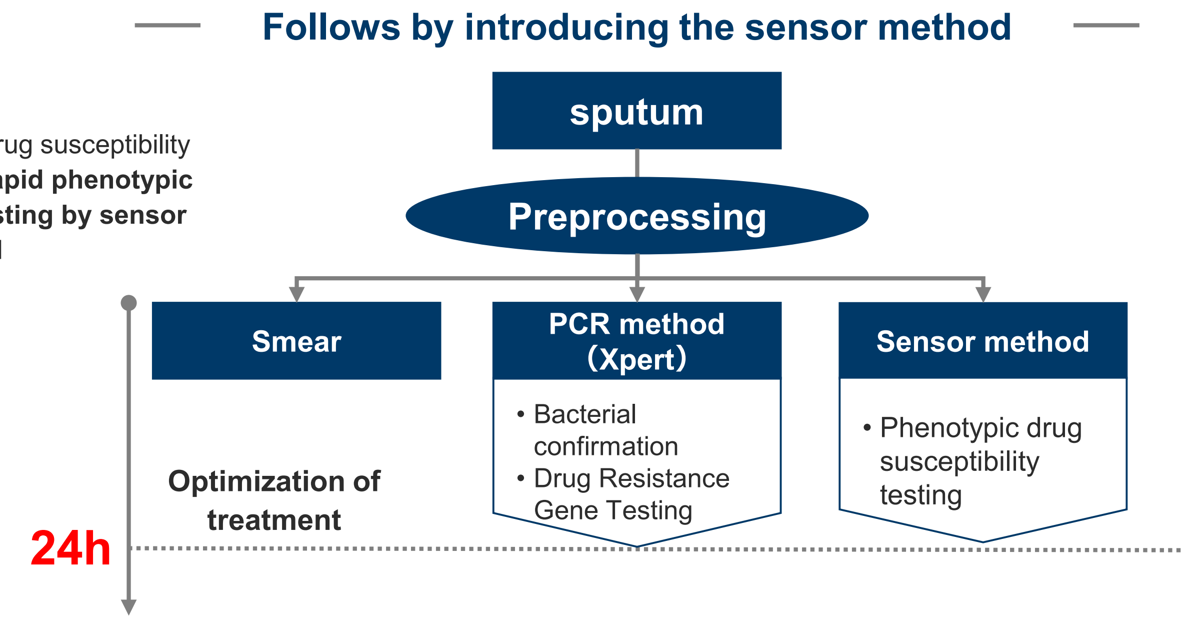

- Phenotypic susceptibility test results for anti-tuberculosis drugs, including INH, can be obtained at the initial treatment stage
- Unlike the PCR method, there is no need to target each gene for testing.
- The techniques such as culture methods are not required, and the time to determine results can be greatly reduced

- The PCR method has poor other than RFP resistance
- In the PCR method, it is necessary to target each gene and carry out the test
- The PCR method cannot directly reflect the actual clinical efficacy in some cases.
- The culture method requires technique, and it takes time to judge the results.
