# Supplementary material for: Near-field sensor array with 65-GHz CMOS oscillators can rapidly and comprehensively evaluate drug susceptibility of Mycobacterium
Source: Sci Rep. 2023 Mar 7;13:3825. doi: 10.1038/s41598-023-30873-9 (PMC9990582; doi:10.1038/s41598-023-30873-9)
Supplement: Supplementary file 8 — Supplementary Table 1. [file 41598_2023_30873_MOESM8_ESM.pdf]

| Drug | Abbreviation | Solvent | Final concentration | CAS No. | Manufacturer ID | Manufacturer |
|---|---|---|---|---|---|---|
| Refampicin | RFP | 100% Methanol | * 1.0 or 2.0 μg/mL | 13292-46-1 | 189-01001 | FUJIFILM Wako Chemicals |
| Isoniazid | INH | Ultrapure water | * 0.1 or 0.2 μg/mL | 54-85-3 | 1349706 | FUJIFILM Wako Chemicals |
| Pyrazinamide | PZA | Ultrapure water | * 100.0 or 50.0 μg/mL | 98-96-4 | 195473 | FUJIFILM Wako Chemicals |
| Streptomycin | SM | Ultrapure water | * 1.0 or 2.0 μg/mL | 3810-74-0 | 190-14342 | FUJIFILM Wako Chemicals |
| Ethambutol | EB | Ultrapure water | * 5.0 or 10.0 μg/mL | 1070-11-7 | E889800 | FUJIFILM Wako Chemicals |
| Levofloxacin | LVFX | 0.1 N Hydrochloric acid | 1.0 or 2.0 μg/mL | 100986-85-4 | BIL1009 | FUJIFILM Wako Chemicals |
| Kanamycin | KM | Ultrapure water | 2.5 or 5.0 μg/mL | 25389-94-0 | 08976-71 | NACALAI TASQUE, INC |
| Enviomycin | EVM | Ultrapure water | 5.0 or 10.0 μg/mL | 33103-22-9 | 4987-153-08503-9 | asahikasei-pharma |
| Ethionamide | TH | 100% Methanol | 2.5 or 5.0 μg/mL | 536-33-4 | E890420 | FUJIFILM Wako Chemicals |
| Cycloserine | CS | Ultrapure water | 32.0 or 64.0 μg/mL | 68-41-7 | 030-21003 | FUJIFILM Wako Chemicals |
| Delamanid | DLM | Dimethyl sulfoxide | 0.06 or 0.12 μg/mL | 681492-22-8 | S5007 | Selleck |
| Bedaquiline | BDQ | Dimethyl sulfoxide | 1.0 or 2.0 μg/mL | 845533-86-0 | S4854 | Selleck |
| Ciprofloxacin | CPFX | 0.1 N Hydrochroric acid | 1.25 or 2.5 μg/mL | 85721-33-1 | 17850 | Sigma-Aldrich |

* equivalent to CDC recommended critical concentration for Mycobacterium tuberculosis
